# Stable isotope labeling and ultra-high-resolution NanoSIMS imaging reveal alpha-synuclein-induced changes in neuronal metabolism in vivo

**DOI:** 10.1186/s40478-023-01608-8

**Published:** 2023-09-29

**Authors:** Sofia Spataro, Bohumil Maco, Stéphane Escrig, Louise Jensen, Lubos Polerecky, Graham Knott, Anders Meibom, Bernard L. Schneider

**Affiliations:** 1 Brain Mind Institute, Ecole Polytechnique Fédérale de Lausanne (EPFL), Lausanne, Switzerland; 2 Laboratory for Biological Geochemistry, Ecole Polytechnique Fédérale de Lausanne (EPFL), Lausanne, Switzerland; 3 Department of Earth Sciences, Faculty of Geosciences, Utrecht University, Utrecht, The Netherlands; 4 Bioelectron Microscopy Core Facility, Ecole Polytechnique Fédérale de Lausanne (EPFL), Lausanne, Switzerland; 5 Center for Advanced Surface Analysis, Institute of Earth Sciences, University of Lausanne, Lausanne, Switzerland; 6 Bertarelli Platform for Gene Therapy, Ecole Polytechnique Fédérale de Lausanne (EPFL), Geneva, Switzerland; 7 EPFL SV PTECH PTBTG, Ch. Des Mines 9, 1202 Geneva, Switzerland; 8 EPFL ENAC IIE LGB, Station 2, 1015 Lausanne, Switzerland

**Keywords:** Parkinson’s disease, Rodent model, Substantia nigra, Alpha-synuclein, Glucose metabolism, NanoSIMS, SILK-SIMS

## Abstract

In Parkinson’s disease, pathogenic factors such as the intraneuronal accumulation of the protein α-synuclein affect key metabolic processes. New approaches are required to understand how metabolic dysregulations cause degeneration of vulnerable subtypes of neurons in the brain. Here, we apply correlative electron microscopy and NanoSIMS isotopic imaging to map and quantify ^13^C enrichments in dopaminergic neurons at the subcellular level after pulse-chase administration of ^13^C-labeled glucose. To model a condition leading to neurodegeneration in Parkinson’s disease, human α-synuclein was unilaterally overexpressed in the *substantia nigra* of one brain hemisphere in rats. When comparing neurons overexpressing α-synuclein to those located in the control hemisphere, the carbon anabolism and turnover rates revealed metabolic anomalies in specific neuronal compartments and organelles. Overexpression of α-synuclein enhanced the overall carbon turnover in nigral neurons, despite a lower relative incorporation of carbon inside the nucleus. Furthermore, mitochondria and Golgi apparatus showed metabolic defects consistent with the effects of α-synuclein on inter-organellar communication. By revealing changes in the kinetics of carbon anabolism and turnover at the subcellular level, this approach can be used to explore how neurodegeneration unfolds in specific subpopulations of neurons.

**Supplementary Information:**

The online version contains supplementary material available at 10.1186/s40478-023-01608-8.

## Introduction

Parkinson’s disease (PD) is a progressive neurodegenerative disease that affects the function and survival of neuronal populations in various brain regions, with a pattern reflecting the selective vulnerability of different neuron subtypes. In the basal ganglia, dopaminergic neurons located in the *substantia nigra pars compacta* (SNpc) are among the neuronal subtypes that are most vulnerable to the disease. Furthermore, the loss of nigrostriatal dopaminergic innervation is the main cause of the motor symptoms that characterize PD.

Idiopathic PD is a multifactorial disease linked to genetics, the environment, and aging. Although the cause of sporadic PD remains poorly understood, the identification of genetic factors associated with familial forms of the disease, such as multiplication of the *SNCA* gene, together with the presence of Lewy bodies containing α-synuclein (α-syn) fibrils, point to α-syn accumulation, misfolding and aggregation as a core mechanism in PD pathogenesis. Alpha-syn dyshomeostasis is known to profoundly affect the metabolic activity and function of neurons via a plethora of cellular mechanisms. When aberrantly localized in the neuronal cell body, α-syn interacts with multiple membranous organelles and thereby affects inter-organellar communication and cellular trafficking [3, 28, 47]. In pathogenic conditions, α-syn impairs mitochondrial function [13] as well as vesicle trafficking in the secretory pathway [20] via inhibition of vesicle transfer from the endoplasmic reticulum (ER) to Golgi apparatus [11]. Alpha-syn also affects the function of autophagosomes and autolysosomes [33, 34]. Ultimately, the deposition of α-syn fibrils into Lewy bodies leads to organelle sequestration, further compromising cellular functions [32, 48]. Other mechanisms, such as the α-syn-induced impairment of ER-mitochondria signaling, have recently been highlighted [21]. However, it remains unclear how these defects affect core cellular functions to progressively lead to the degeneration of vulnerable neuronal subtypes as observed in PD. Notably, little is known about associated pathological changes in cell metabolism at the level of neuronal compartments and key organelles.

Identifying brain metabolic defects at subcellular level in vivo requires dedicated techniques. One such technique combines stable isotope labeling kinetics with secondary ion mass spectrometry (SILK-SIMS) [41]. The NanoSIMS is an ion microprobe instrument capable of imaging and quantifying elemental and isotopic variations in biological tissues with about 100 nm lateral resolution, following sample preparation similar to that required for electron microscopy [27, 38]. The resulting isotope maps can be precisely correlated with scanning electron microscopy (SEM) images of cellular ultrastructure allowing quantification of the isotopic composition at a level of subcellular compartments such as the nucleus, nucleolus, mitochondria, and Golgi apparatus. Combined with stable isotope labeling experiments using, e.g., mice and rats, the correlative NanoSIMS and SEM imaging has proven an efficient method to study metabolic turnover of brain tissue in normal homeostasis, as well as in brain disease models [7, 30, 31, 35, 51, 52].

The aim of the present study was to develop a method to gain new insights into α-syn-induced pathological changes in cell metabolism at the level of compartments and organelles within nigral dopaminergic neurons. To this end, we combined a ^13^C-glucose pulse-chase experiment with correlative SEM and NanoSIMS imaging and quantified the effects of α-syn overexpression on anabolic incorporation and turnover of glucose-derived carbon in major compartments of the neuronal cell body (cytoplasmic versus nuclear), as well as in specific organelles (mitochondria, Golgi apparatus and lysosomes). Our data revealed specific changes in ^13^C enrichments in nigral neurons following α-syn overexpression compared with their healthy counterparts: neuronal cell bodies exhibited increased overall carbon turnover, with a decrease in relative carbon incorporation in the nuclear compartment. At the level of organelles, we observed a lower carbon incorporation in the mitochondria as well as lower carbon turnover in the secretory Golgi apparatus.

## Materials and methods

### Rat model of Parkinson’s disease

To induce pathogenic α-syn overexpression in the ventral midbrain, 11 rats were injected in the SN with serotype 6 adeno-associated viral (AAV) vectors coding for the human wild-type α-syn protein (nucleotides 46–520, NM_000345), as previously described [5]. In this vector, the expression of the human α-syn protein is under the control of the mouse phosphoglycerate kinase 1 (pgk1) constitutive promoter (AAV2/6-pgk1:β-globin-intron:α-syn:WPRE). The coordinates used for stereotactic injection were: − 5.2 mm (anteroposterior), − 2 mm (mediolateral), − 7.8 mm (dorsoventral, relative to skull surface), − 3.3 mm (tooth bar). Viral vectors were produced and titrated as previously described [16]. Briefly, relative AAV infectivity was determined by real-time PCR (rtPCR) quantification of double-stranded vector genomes present in total DNA isolated from HEK293 cells, 48 h post-transduction. The infectivity rate expressed in ‘transducing units’ (TU) was calculated according to a known infectivity of a standard virus encoding GFP (AAV2/6-cmv-eGFP), whose titer was estimated via flow cytometry in similar conditions. Both the vector expressing α-syn (AAV-α-syn) and the control non-coding vector were used at a total injected dose of 1.5 × 10^7^ TU in a volume of 2 µL. The AAV-α-syn vector was injected in the right hemisphere and the control vector in the left hemisphere of the same animal.

Animal handling and experimentation used in this study were performed according to the Swiss legislation and the European Community Council directive (86/609/EEC) for the care and use of laboratory animals. The protocol was approved by the local ethics committee and by the veterinary authorities. The experiments were performed using adult female Sprague–Dawley rats (Janvier) weighing around 200 g. During the whole experiment, the animals were maintained on 12 h light–dark cycle and fed ad libitum with standard chow. Animals were maintained in standard housing conditions for a period of 30 days post-injection until α-syn overexpression induced degenerative effects. At that time, the animals were subjected to labelling with uniformly ^13^C-labeled glucose, as detailed below. Animals were sacrificed and brain tissues were subsequently processed for SEM and NanoSIMS analyses.

### ^13^C glucose labelling

**Fig. 1:**
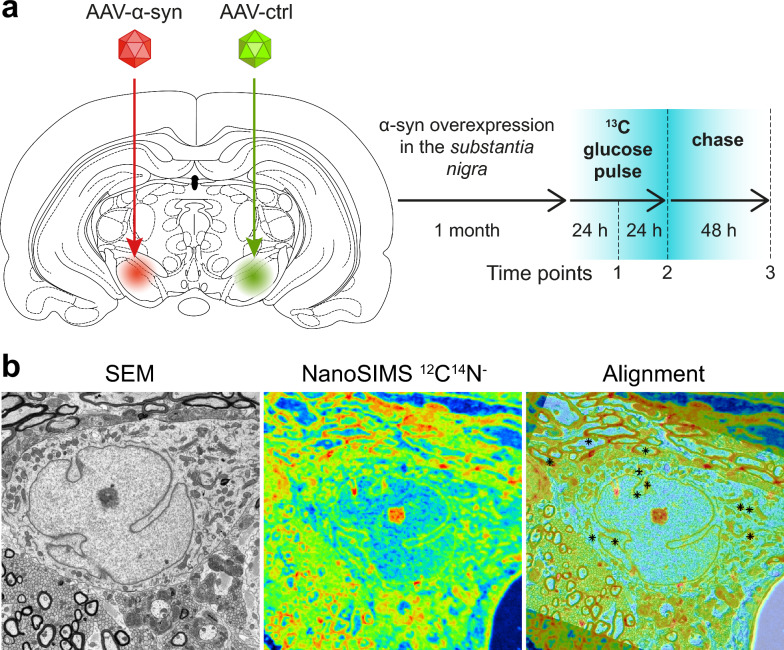
SILK-SIMS measurement of ^13^C labeling in a model of induced α-syn overexpression. **a** Pathogenic conditions were induced in the rat ventral midbrain by unilateral AAV-mediated α-syn overexpression for one month. Subsequently, the animal was subjected to a 48 h pulse with ^13^C-labeled glucose administered in the drinking water. The effects of α-syn overexpression on the kinetics of ^13^C incorporation were determined by comparing the brain hemisphere injected with AAV6-α-syn with the control hemisphere injected with a non-coding AAV6 vector. **b** Representative example showing how the SEM ultrastructure image was aligned with the isotopic image measured by NanoSIMS. Note that the NanoSIMS image was rotated relative to the SEM image and acquired with a lower lateral resolution (about 15-fold). Additionally, the NanoSIMS image was slightly distorted (stretched or squeezed) relative to the SEM image because of the movement of the sample stage caused by temperature variations during the relatively long NanoSIMS measurement. The alignment of the two images was done by matching the locations of multiple reference points manually defined by the user. In the ‘alignment’ image, points indicated by ‘ + ’ and ‘**✕**’ were defined in the SEM and NanoSIMS image, respectively. Superimposed reference points are indicated with ❊.

During the pulse phase starting 30 days post-vector injection, 5% (w/v) ^13^C-labeled glucose (D-Glucose-^13^C_6_, ^13^C atom fraction of 99%, Sigma-Aldrich, Switzerland) was added to the drinking water. The amount of glucose-supplemented water ingested by each individual rat was regularly measured (every 12 h) and was found to be very similar among animals (58.3 ± 2.0 mL per day), also when taking into account body weight. Animals were initially divided into 3 groups containing 3–4 animals per group based on the ^13^C pulse-chase protocol. Groups 1 and 2 ingested the ^13^C labeled glucose for 24 h and 48 h before sacrifice, respectively (pulse phase), whereas Group 3 first ingested ^13^C labeled glucose for 48 h, followed by ingestion of isotopically normal glucose (added at a concentration of 5% (w/v) in drinking water) for additional 48 h (chase phase), after which they were sacrificed for analysis (Fig. 1a). Group 3 contained only 3 rats after one animal died during stereotaxic surgery.

### Tissue preparation

At each sampling time point, animals were deeply anaesthetized via inhalation of isofluorane, and, as soon as the breathing stopped, they were immediately perfused via the heart with 10 mL of isotonic PBS at the speed of 60 mL/min, followed with 300 mL of a buffered mixture of 2.5% glutaraldehyde and 2% paraformaldehyde (0.1 M phosphate buffer (PB), pH 7.4). After the perfusion was complete the animal was left for 2 h. The brain was then removed from the skull and 80 µm thick, coronal sections were cut through the frontal striatum and the SN regions using a vibratome (Leica VT1200S; Leica Microsystems, Vienna, Austria).

The vibratome sections were washed in cacodylate buffer (0.1 M, pH 7.4) and then stained with 1% osmium tetroxide and 1.5% potassium ferrocyanide for 40 min. This was immediately followed by further staining in 1% osmium tetroxide alone for another 40 min. After washing in double-distilled water, the sections were stained again with 1% aqueous uranyl acetate for 40 min. Then, the sections were dehydrated in graded alcohol series (2 × 50% ethanol, 1 × 70%, 1 × 90%, 1 × 95%, 2 × 100%: 3 min each change), and gradually infiltrated with Durcupan resin (Fluka, Buchs, Switzerland) in ethanol at 1:2, 1:1 and 2:1 ratios for 30 min each. Finally, the sections were infiltrated twice with pure Durcupan resin for 2 × 30 min followed by fresh Durcupan for 4 h. The sections were flat embedded between glass slides in fresh resin and polymerized for at least 24 h inside an oven set at 65 °C.

### Sample preparation for histology and SEM imaging

Once the resin had hardened, the region of *striatum* and *substantia nigra* (A9 region) from both the left (injected with the control non-coding vector) and right (injected with AAV-α-syn) hemispheres were identified according to the rat brain atlas (Paxinos and Watson, 1996), cut out and glued with acrylic cement onto a resin block. Then, 0.5 µm semithin sections were cut using an ultramicrotome (Leica UC6, Leica Microsystems, Vienna, Austria) equipped with a diamond knife (Diatome, Switzerland). These sections were collected onto round silicon wafers (10 mm diameter) and lightly gold-coated (a few nm thickness). These sections were used for SEM and NanoSIMS imaging. Neuronal bodies in the *pars compacta* of the SN were imaged with a Zeiss Gemini 500 Field Emission Scanning Electron Microscope (Zeiss, Oberkochen, Germany). Micrographs (4096 × 3072 pixels) were recorded by using an in-lens energy selective backscatter detector (EsB) designed to enhance material contrast at low-kV imaging. The backscattered electrons were energy filtered with the EsB grid in front of the detector tuned to 1500 V and the accelerating voltage was 3 kV. The micrographs were recorded with inverted contrast directly. SEM images of individual neurons were used to analyze the aspect ratios of mitochondria (length-over-width) as well as the frequency of the observed contacts between the mitochondrial outer membrane and the ER (MERC) using the Fiji software.

**Fig. 2:**
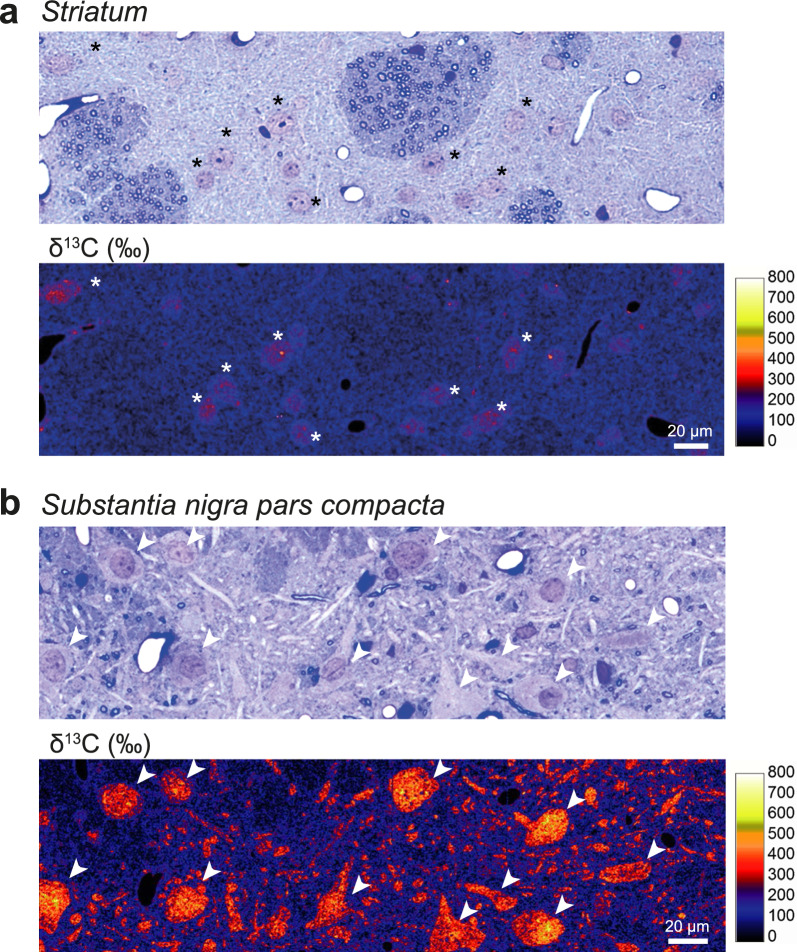
Carbon isotope labelling in the brain following 48 h pulse with ^13^C-labeled glucose: comparison of the *striatum* and *substantia nigra pars compacta*. **a** Representative light microscopy image of the striatal tissue showing a toluidine blue-stained semi-thin section (upper panel), adjacent to a map of ^13^C isotope enrichment in the same brain region (lower panel). Note the weak ^13^C labeling mainly present in neuronal cell bodies (*). **b** Representative light microscopy image of the *substantia nigra pars compacta* showing large neuronal cell bodies and neurites with intense ^13^C enrichment (arrowheads). To facilitate direct comparison, the same color-coded δ^13^C (‰) intensity scale is applied in (**a**) and (**b**). Scale bars: 20 µm

For the images of the *striatum* and SNpc (examples shown in Fig. 2), semi-thin sections were stained with 1% toluidine blue stain, and imaged using a light microscope (Nikon) equipped with a 20× objective (Olympus AX70). These sections were subsequently imaged by NanoSIMS as described below. Other NanoSIMS imaging was performed on semi-thin sections placed on silicon wafers.

### NanoSIMS isotopic imaging

To quantify the ^13^C enrichment in neuronal bodies with a subcellular resolution and correlate it with the ultrastructure information gained via SEM, the exact same regions were imaged with a NanoSIMS 50L ion microprobe using Cs^+^ primary ion beam. After SEM imaging and prior to NanoSIMS analysis, the same silicon wafers containing sections were coated with an additional 10–15 nm of gold. Following pre-sputtering for 5 min with a primary beam of about 10 pA to remove the metal coating, the beam was focused to a spot size of about 150 nm (1–3 pA) and rastered across an area of typically 50 × 50 µm^2^ (256 × 256 pixels) with a pixel dwell time of 5 ms. Secondary ions ^12^C^14^N^−^ and ^13^C^14^N^−^ (that yielded the highest count rates) were counted simultaneously (multi-collection) in electron multipliers at a mass resolution sufficient to avoid all potentially problematic mass-interferences [51, 52]. The rastering was repeated up to 15 times over the same area to increase the overall ion counts and thus improve the precision of the ^13^C enrichment quantification. For higher resolution imaging, the primary beam was focused to about 100 nm and images of regions 20 × 20 µm^2^ (256 × 256 pixels) in size were obtained with the raster repeated 10 times.

### NanoSIMS data processing

NanoSIMS data were processed using the L’IMAGE^®^ software (Larry R. Nittler, Carnegie Institution of Washington, Washington, DC, USA). First, individual ion count images acquired during each raster were aligned and accumulated. Subsequently, images of the ^13^C/^12^C isotope ratio were obtained by taking the ratio between the accumulated ^13^C^14^N^−^ and ^12^C^14^N^−^ ion count images. The ^13^C enrichment is reported in the δ notation, as parts per thousand deviation from the ^13^C^14^N^−^/^12^C^14^N^−^ ratio measured on similar brain tissues with a natural carbon isotopic composition:

$$\delta^{13} {\text{C}}(\permil) = \left( {\frac{{R_{mes} }}{{R_{nat}}} - 1} \right) \times 1000,$$

where R_mes_ and R_nat_ are the ^13^C^14^N^−^/^12^C^14^N^−^ ratios measured in samples obtained from experiments with ^13^C-labeled and ^13^C-unlabelled (natural) glucose, respectively, both prepared and analyzed under identical conditions.

NanoSIMS data were additionally processed with the Look@NanoSIMS software [39], which was modified to allow precise alignment of the ultra-high resolution SEM image and the corresponding NanoSIMS image and thus extract the isotopic compositions of small structures that are not identifiable solely based on the NanoSIMS image. Steps required to perform this analysis, which involve resampling of the NanoSIMS image, alignment with the SEM image, drawing of ROIs based on the SEM image, and quantification of secondary ion counts and ion count ratios in these ROIs mapped to the original NanoSIMS image, are described in detail in the Additional file 1.

### Statistical analysis

Statistical analyses were performed with the GraphPad Prism software. Numerical data are reported as mean ± SD. Violin plots of numerical data show the median as well as upper and lower quartiles. The statistical tests as well as the number of replicates are indicated in the figure legends. A non-parametric Mann–Whitney test was applied to data sets for which the distribution of the values did not pass normality testing. The α level of significance was set at 0.05.

## Results

Metabolism and turnover of macromolecules and organelles within tissues can be explored using techniques based on the incorporation of stable isotopes. In particular, the anabolic metabolism of ^13^C-labeled glucose leads to the production of metabolites incorporated into proteins, lipids, and nucleic acids, i.e., the building blocks of the supramolecular cellular structures. In the central nervous system, ^13^C-labeling in neurons following administration of ^13^C glucose on the time scale of hours and days can be detected using NanoSIMS imaging in aldehyde-fixed brain tissues [51]. Here, we investigated the incorporation and turnover of glucose-derived carbon in specific sub-populations of neurons in the adult rat brain following a pulse-chase experiment with ^13^C-labeled glucose (Fig. 1). In particular, we applied this approach to an animal model of PD based on human wild-type α-syn overexpression, to explore how the pathogenic accumulation of this protein affects carbon metabolism in nigral neurons at subcellular level.

Local overexpression of the human α-syn protein in the rat ventral midbrain was induced by unilaterally injecting an AAV6-α-syn vector in the SN of adult rats (Fig. 1a). In this model system, the overexpression of human α-syn was confined to the injected hemisphere and the contralateral hemisphere could be used as a control to identify the effects of α-syn on glucose-derived carbon metabolism in the affected brain tissue. In order to control for potential effects of vector injection, the same dose of a non-coding AAV6 vector was injected in the contralateral hemisphere. As previously shown, overexpression of human α-syn in nigral dopaminergic neurons leads to progressive and selective degeneration of dopaminergic neurons over 1–4 months, loss of nigrostriatal dopamine innervation, and reduced dopamine release in the *striatum* [5, 19].

To explore the early consequences of human α-syn on ^13^C incorporation in neurons, we compared quantitative NanoSIMS images of ^13^C/^12^C ratios throughout a pulse-chase experiment, one month after AAV6-α-syn injection. Glucose uniformly labeled with ^13^C was added to the drinking water during a 48 h pulse phase. The chase period was carried out between 48 and 96 h, during which the animals received drinking water supplemented with the same concentration of isotopically normal glucose. The level of ^13^C enrichment was assessed in brain tissues from the *striatum* and the SN, at three time points: during (at 24 h; N = 3 rats) and at the end of the pulse phase (at 48 h; N = 4 rats), as well as after the chase phase (at 96 h; N = 3 rats) (Fig. 1a). At each time point, we correlated the SEM image of the ultrastructure of the brain tissue with the NanoSIMS isotopic maps (Fig. 1b). The NanoSIMS ^12^C^14^N^−^ map (which allows identification of many subcellular structures) was first used to obtain a precise correlation between NanoSIMS and SEM images using the Look@NanoSIMS software [45], as illustrated in Fig. 1b. It was then possible to precisely map and measure ^13^C enrichment in the brain tissue, at both cellular and subcellular levels.

### ^13^C-labeling of neurons in the basal ganglia reveals clear differences in anabolic carbon incorporation

PD is characterized by the selective vulnerability of dopaminergic neurons located in the SNpc. As these neurons have an extremely complex axonal arborization and make numerous synaptic contacts in the *striatum*, it is anticipated that they have a very high metabolic turnover [6, 44]. The resulting high metabolic activity may represent a significant stress, which has been proposed to make nigral neurons particularly vulnerable to the development of PD [39, 50].

To compare the metabolic activity of two major neuron populations in the basal ganglia, we assessed the level of ^13^C incorporation in the *striatum* and SNpc at the end of the pulse phase (Fig. 2). In both brain regions, ^13^C accumulation was most evident in neuronal cell bodies. When comparing the *striatum* and the SNpc, ^13^C enrichment was distinctly lower in the *striatum*, where GABAergic medium spiny neurons represent the most abundant neuronal population, exhibiting ^13^C enrichment values in the range of 100–300 ‰ mainly confined to the neuronal nuclei (Fig. 2a). In contrast, ^13^C enrichment in the neuronal cell body was on average about 1.8-fold higher in the SNpc, primarily localized to a population of large neurons with a morphology similar to dopaminergic neurons (Fig. 2b), which represent 70% of the neurons in this brain region [15, 37]. In these neurons, ^13^C enrichment ranged between 200 and 500‰ distributed throughout their entire neuronal cell bodies and neurites, confirming their relatively high anabolic activity. We focused our subsequent analyses on this population of neurons, assessing the ^13^C enrichment in the neuronal cell bodies as well as specific cellular sub-compartments and organelles to determine the effects of human α-syn overexpression.

### Alpha-synuclein overexpression enhances overall ^13^C turnover in nigral neurons

**Fig. 3:**
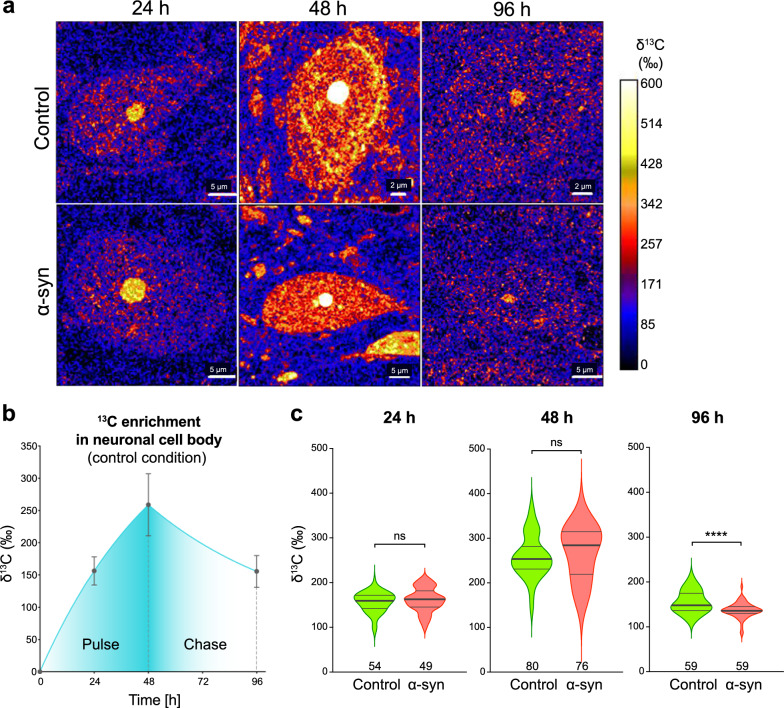
Kinetics of ^13^C incorporation in neuronal cell bodies in the *substantia nigra pars compacta*. **a** Representative maps of ^13^C isotope enrichment throughout pulse-chase experiment in neurons located in the SNpc, either in the control or AAV6-α-syn-injected SNpc. Scale bars: 2 and 5 µm. **b** Pulse-chase experiment: quantification of ^13^C enrichment in the whole cell body of nigral neurons in the SNpc injected with the non-coding control vector. Pulse phase: ^13^C enrichment at 24 h and 48 h after starting ^13^C-labeled glucose administration in the drinking water. The graph shows curve fitting with exponential plateau. Chase phase: an additional measurement was performed at 96 h and curve fitting was based on the assumption that the observed drop in ^13^C-enrichment follows a one-phase exponential decay. Data represent mean ± SD. **c** Violin plots showing ^13^C enrichment in the whole cell body. The graphs compare nigral neurons located in the SNpc injected with a non-coding AAV6 vector to their counterparts in the contralateral AAV6-α-syn-injected SNpc. The pulse-chase experiment was performed 30 days after intranigral vector injection. Note the effects of human α-syn overexpression on carbon turnover in the neuronal cell bodies during the chase period. The thick lines in the violin plots represent the median and the thin lines the upper and lower quartiles. The number of neurons analyzed is indicated at the bottom of the histogram bar. 24 h: N = 3 animals per group; 48 h: N = 4 animals per group; 96 h: N = 3 animals per group. Statistical analysis: multiple Mann–Whitney tests with False Discovery Rate (FDR) approach; ns = not significant; **** FDR-adjusted *P* < 0.0001

Large neuronal cell bodies present in the SNpc were outlined using NanoSIMS ^12^C^14^N^−^ maps and average levels of ^13^C enrichment were measured within areas covering individual cell bodies from a randomly chosen population of 49–80 neurons per condition (Fig. 3). In the control condition, we observed a progressive increase in the anabolic ^13^C enrichment during the pulse phase, reaching on average 156 ± 22‰ and 259 ± 48‰ at 24 h and 48 h, respectively (Fig. 3a,b). At the end of the chase phase, i.e. 48 h after administration of ^13^C-labeled glucose was stopped and replaced by the administration of unlabeled glucose, the enrichment declined by − 40% to 156 ± 25‰ (Fig. 3a,b).

Next, we assessed the effects of overexpressing the human α-syn protein on the glucose-derived carbon turnover by comparing nigral neurons located in the AAV6-α-syn injected hemisphere (pathological condition) to those located in the contralateral control hemisphere (Fig. 3a,c). Overall, the level of ^13^C enrichment was similar for both populations of neurons during the entire pulse period, indicating that α-syn overexpression had no major effects on the overall glucose-derived carbon incorporation in neurons (Fig. 3a,c). However, a steeper decline in average ^13^C enrichment was observed during the chase phase (96 h time point) in the pathological condition. At the end of the chase period, ^13^C enrichment had declined by 49%, to an average value of 136‰, which was significantly different from the control hemisphere (Fig. 3c). NanoSIMS imaging of specific neuronal populations therefore revealed changes in the turnover of the ^13^C-labeled macromolecular content, which can be ascribed to α-syn-induced pathogenic conditions in the brain.

### Cytoplasmic and nuclear compartments of nigral neurons are differentially affected by α-synuclein overexpression

**Fig. 4:**
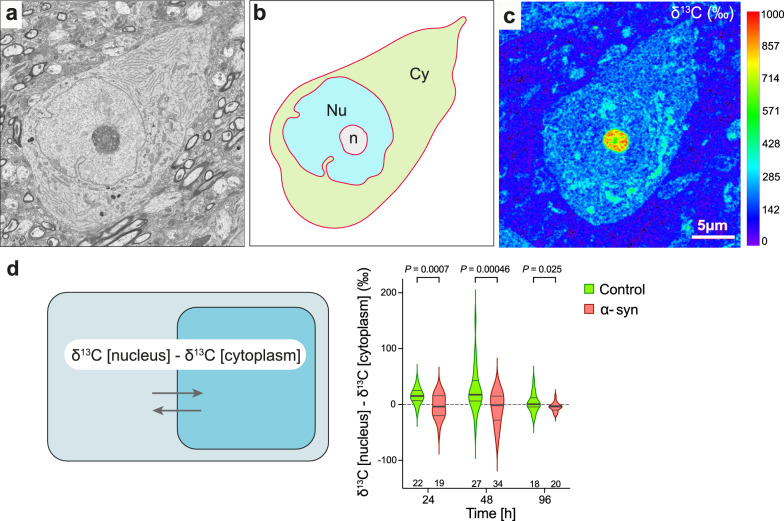
Effect of human α-syn overexpression on glucose-derived carbon incorporation and turnover in the nuclear versus cytoplasmic compartment. **a** Representative SEM image of a large neuronal cell body located in the SNpc. **b** Segmentation of the SEM image into cytoplasmic (Cy), nuclear (Nu), and nucleolar (n) compartments. Outlines of the compartments were drawn manually using the Look@NanoSIMS software (see Additional file 1). **c** The mask generated in (**b**) was used to measure ^13^C enrichment in the nuclear versus cytoplasmic compartments in the corresponding isotopic NanoSIMS maps. Note that neurons were excluded from the analysis when the highly ^13^C enriched nucleolar compartment was present in the image. **d** The schematic illustrates the determination of the difference in ^13^C enrichment between the nuclear and cytoplasmic compartments. The graph compares neuronal cell bodies located in the control and α-syn-overexpressing SNpc at the 24 h, 48 h (pulse) and 96 h (chase) time points. Note the loss of nuclear glucose-derived ^13^C enrichment in the α-syn overexpression condition during the pulse period. The thick lines in the violin plots represent the median and the thin lines the upper and lower quartiles. The number of neurons analyzed is indicated at the bottom of the histogram bar. 24 h: N = 3 animals per group; 48 h: N = 4 animals per group; 96 h: N = 3 animals per group. Statistical analyses: multiple unpaired two-tailed t-test with False Discovery Rate (FDR) approach; FDR-adjusted *P* values are indicated in the graph. Scale bar: 5 µm

Next, we extended our analysis to investigate the effects of α-syn overabundance in major subcellular compartments, the cytoplasm and nucleus, by correlating NanoSIMS isotopic maps with ultra-high resolution SEM images of sections of individual large-sized nigral neurons (Fig. 4a–c). The cytoplasmic and nuclear compartments were delineated in the cell body to determine their mean ^13^C enrichment. As the nucleoli were typically highly enriched in ^13^C, they strongly increased the average ^13^C enrichment among those individual nuclei. Therefore, if the nucleolus was visible within a cell section, the neuron was excluded from the analysis. In each neuron, the difference between ^13^C enrichments in the nuclear and cytoplasmic compartments was determined, to normalize away potential differences in ^13^C incorporation observed among neurons (Fig. 4d). In the control condition, ^13^C enrichments in the nucleus was on average 15‰ and 25‰ higher than in the cytoplasm at the 24 and 48 h time points, respectively (Fig. 4d). Remarkably, this difference between the compartments nearly disappeared in the neurons analyzed in the pathological condition (Fig. 4d), indicating that α-syn overexpression affected the cytoplasmic versus nuclear anabolic activity. At the 96 h time point, the difference in ^13^C enrichment between the cytoplasm and the nucleus nearly disappeared in both the α-syn and control neurons (Fig. 4d).

### Correlative NanoSIMS/SEM imaging of nigral neurons revealed the kinetics of glucose-derived carbon incorporation at the level of organelles

**Fig. 5:**
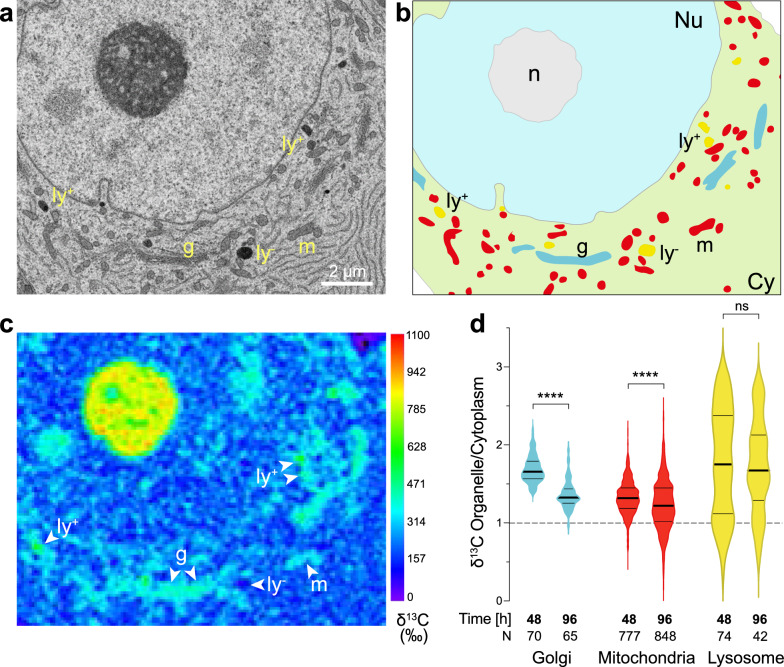
NanoSIMS analysis of ^13^C enrichment in specific organelles within the cell body of nigral neurons (control hemisphere). **a** Representative SEM image of a large neuronal cell body located in the SNpc allowed to identify three types of organelles, the mitochondria (m), the Golgi apparatus (g) and lysosomes (ly). **b** SEM-based segmentation of compartments and organelles in the cell body; Cy: cytoplasm; Nu: nucleus; n: nucleolus. **c** Corresponding map of ^13^C enrichment. Note the presence of both ^13^C-rich lysosomes (ly^+^) and ^13^C-poor lysosomes (ly^−^) within the same neuronal cell body. **d** ^13^C enrichment in Golgi, mitochondria and lysosomes, as compared to the cytoplasm, measured at the end of the pulse with ^13^C-labeled glucose (48 h time point) and after the chase period (96 h time point). Note the significant decrease in ^13^C enrichment in the Golgi and mitochondria. In contrast, ^13^C enrichment in lysosomes is highly variable and does not significantly decrease over the chase period. The thick lines in the violin plots represent the median and the thin lines the upper and lower quartiles. The number of organelles analyzed is indicated at the bottom of the histogram bar. 48 h: N = 11 neurons from 2 rats; 96 h: N = 12 neurons from 3 rats. Statistical analysis: Mann–Whitney two-tailed test; ns = not significant; ****P < 0.0001. Scale bar: 2 µm

Next, SEM ultrastructural imaging was used to delineate populations of organelles in the cell body of large-sized neurons located in the control SNpc. The analysis focused on the Golgi apparatus, mitochondria, and lysosomes (Fig. 5). For each of these organelle populations, a ROI outline was generated based on the SEM image, which was then applied to the corresponding precisely aligned ^13^C/^12^C map to determine the ^13^C enrichment in each organelle (Fig. 5b, c). The organellar ^13^C enrichments were normalized to the ^13^C enrichment averaged throughout the entire cytoplasm visible within the imaged area of individual neurons, to assess carbon incorporation into organelles relative to the corresponding cytoplasmic ^13^C enrichment levels. This analysis was performed at the 48 h and 96 h time points to investigate potential differences among organelles in the control hemisphere at the end of the pulse phase and during chase (Fig. 5d). At the end of pulse, the average ^13^C enrichments in all organelles were higher than in the cytoplasm (+ 69% in the Golgi apparatus, + 33% in mitochondria and + 79% in lysosomes). Additionally, the labeling of individual lysosomes was highly variable within the same neuron, with some organelles displaying a threefold greater ^13^C enrichment relative to that of the cytoplasm, whereas in others the level of ^13^C enrichment remained very low, even below the average level in the cytoplasm (Fig. 5c, d). Remarkably, during the chase period, we measured the most drastic decline in cytoplasm-normalized ^13^C labeling in the Golgi apparatus (from 1.69 ± 0.17 at 48 h to 1.36 ± 0.18 at 96 h; Fig. 5d). This observation is consistent with the high macromolecule turnover in this organelle due to its involvement in the processing and trafficking or proteins and lipids. In mitochondria, there was also a significant decrease in cytoplasm-normalized ^13^C enrichment during the chase (from 1.32 ± 0.22 to 1.23 ± 0.32). In contrast, the average ^13^C labeling of individual lysosomes remained mostly stable over the chase period. Overall, the kinetics of incorporation and turnover of glucose-derived carbon reveals clear differences in the metabolic activity of these different organelles within neurons.

### Kinetics of glucose-derived carbon in neuronal organelles are differentially affected by α-synuclein overexpression

**Fig. 6:**
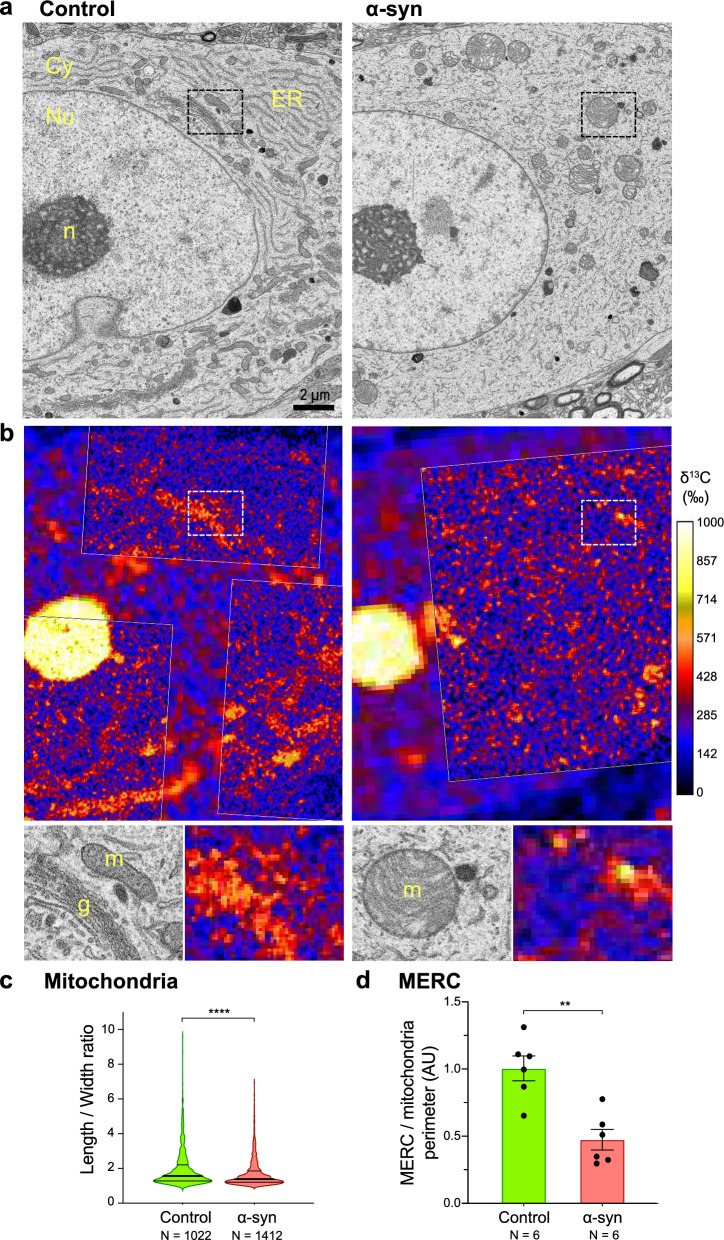
Mapping of ^13^C isotope labeling in three abundant organelles following α-syn overexpression. **a** Representative SEM images of large neuronal cell bodies comparing the SNpc injected with the control non-coding vector (left panel) and the AAV6-α-syn vector (right panel). Note the changes in organelle morphology caused by α-syn overexpression, with fragmented ER and Golgi apparatus and round-shaped mitochondria with abnormal *cristae*. Cy: cytoplasm; Nu: nucleus; n: nucleolus; m: mitochondria; g: Golgi apparatus. **b** Maps of ^13^C isotope enrichment in the corresponding regions of the neuronal cell bodies. Note the regions analyzed at high resolution to determine ^13^C levels in specific organelles. The lower panels show high-magnification images corresponding to the regions indicated by dashed rectangles. Note the low ^13^C enrichment in the mitochondria in the pathologic condition. **c** Measurement of the length-to-width ratio of mitochondria in the neuronal cell bodies, comparing the control and α-syn-overexpressing hemispheres. The thick lines in the violin plots represent the median and the thin lines the upper and lower quartiles. The numbers of mitochondria analyzed from 2 rats per condition are indicated in the x-axis labels. **d** Relative number of mitochondria-ER contacts (MERC) normalized to the total length of mitochondria perimeter (N = 6 neurons from 2 rats per condition). Data represent mean ± standard error (SE). Statistical analysis: Mann–Whitney unpaired two-tailed test; ns = not significant; **P < 0.01; ****P < 0.0001

To determine the effects of α-syn overexpression on the incorporation of glucose-derived carbon in individual organelles, we generated SEM images of nigral neuron cell bodies (Fig. 6a) as well as high-resolution ^13^C enrichment maps (Fig. 6b) in both conditions. These images were aligned as described previously. Overexpression of α-syn in nigral neurons resulted in clear changes in the morphology of major organelles present in the cell body (Fig. 6a). In the contralateral hemisphere injected with the non-coding AAV6 vector, organelle ultrastructure revealed elongated mitochondria as well as ER and Golgi apparatus with typical morphologies. In the pathological condition, however, neuronal cell bodies often displayed a strongly fragmented ER and round-shaped mitochondria with abnormal *cristae*. To confirm the effects of α-syn overexpression on mitochondrial morphology, the length-over-width ratio was quantified for each individual mitochondria and was found to be significantly reduced (i.e., more spherical shape) in the pathological condition as compared to the control hemisphere (Fig. 6c). In addition, we quantified in individual neurons the relative number of contacts between the ER and mitochondria (MERC), normalized to the overall mitochondrial perimeter length. The frequency of MERC was significantly reduced in the pathological condition (Fig. 6d).

**Fig. 7:**
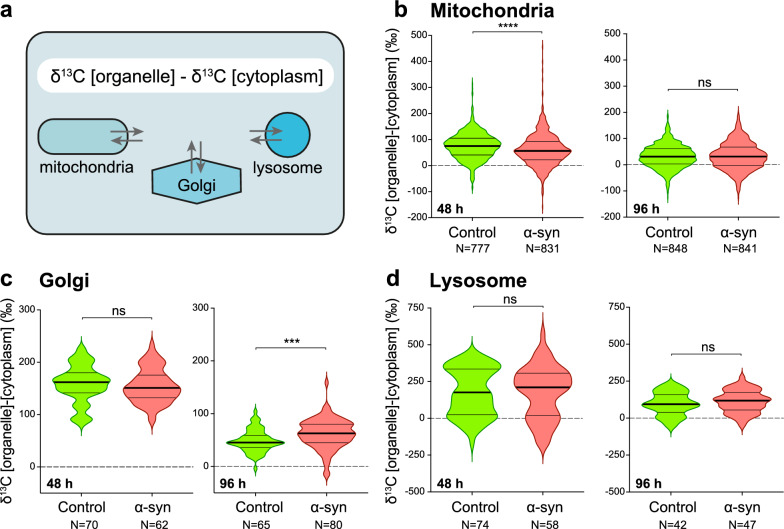
Specific defects caused by human α-syn overexpression in the neuronal cell body are revealed by the kinetics of ^13^C isotope labeling in three abundant organelles. **a** The schematic illustrates the determination of the difference in ^13^C enrichment between three organelle subtypes and the average ^13^C enrichment measured in the cytoplasmic compartment. **b–d** Measured ^13^C enrichments in three organelle subtypes: the mitochondria, the Golgi apparatus and the lysosomes. Violin plots are shown to compare the control and α-syn overexpressing conditions at both the 48 h (pulse) and 96 h (chase) time points. Note the significant decrease in ^13^C incorporation inside mitochondria at 48 h (**b**) and the significantly higher ^13^C labeling of the Golgi apparatus at the end of the chase period (**c**). The thick lines in the violin plots represent the median and the thin lines the upper and lower quartiles. Numbers of organelles analyzed per condition are indicated in the x-axis labels. 48 h: N = 11–12 neurons from 2 rats; 96 h: N = 12 neurons from 3 rats. Statistical analysis: Mann–Whitney unpaired two-tailed test; ns = not significant; ***P < 0.001; ****P < 0.0001

Considering the observed consequences of α-syn on these organelles, we next determined the effects of the α-syn overabundance on ^13^C incorporation and turnover in each organelle population within the neuronal cell bodies. For each organelle subtype, we determined the difference in ^13^C enrichment between the organelle and the average ^13^C enrichment measured in the whole cytoplasm of the corresponding neuron (Fig. 7a–d).

At the end of the pulse period (48 h time point), ^13^C enrichment relative to the cytoplasm was significantly lower in the mitochondria in neurons located in the α-syn-overexpressing hemisphere (median δ^13^C: + 56‰ versus + 75‰ in control SNpc; Fig. 7b). This result indicates a specific effect of α-syn accumulation on the incorporation of glucose-derived carbon into mitochondria. In contrast, there were no significant differences in the levels of ^13^C enrichment measured in the Golgi apparatus and lysosomes between the control and pathological conditions (Fig. 7c, d), indicating that there were no major perturbations of ^13^C incorporation into these organelles.

A similar comparison was performed at the end of the chase period. In populations of mitochondria and lysosomes, there were no significant differences in the levels of ^13^C enrichment between the control and pathological conditions (Fig. 7b, d). However, analysis of the Golgi apparatus revealed another effect of α-syn overexpression. At the 96 h time point (i.e., after the chase phase), the ^13^C enrichment in the Golgi was significantly increased in pathological condition (median δ^13^C: + 63‰) as compared with the control neurons (median δ^13^C: + 45‰) (Fig. 7c). This difference indicates that the α-syn accumulation also affects the function of the Golgi apparatus, most likely by slowing down trafficking of macromolecules to other cellular or extracellular compartments.

## Discussion

The local expression of human α-syn in the SNpc led to alterations of the neuronal metabolism revealed by a specific pattern of differences in glucose-derived ^13^C enrichment in subcellular compartments and organelles. These results demonstrate that the combination of NanoSIMS imaging of stable isotope incorporation and turnover, correlated with SEM to identify subcellular structures, is a powerful approach to illuminate how pathological changes affect neuronal and subcellular functions in the brain in early stages of neurodegeneration.

### The use of SILK-SIMS to explore metabolic defects during neurodegeneration

The molecular signature of neurodegenerative diseases is characterized by the deposition of misfolded proteins in neurons and glial cells. In PD pathogenesis, the overabundance of the α-syn protein has been shown to cause the formation of misfolded oligomers or fibrils, which interact with a range of cellular processes, although the exact sequence of these events remains unclear. Ultimately, the accumulation of α-syn fibrils into intraneuronal inclusions leads to the trapping of defective organelles, coinciding with major perturbations of cellular organization [32, 48], which can affect metabolic activity in the affected neurons. Here, we used AAV-mediated overexpression of the α-syn protein to induce progressive toxic effects in nigral dopaminergic neurons. In this animal model, α-syn mainly accumulates as disordered monomers [17]. Three to four months after vector injection, misfolded α-syn is detectable using the 5G4 antibody [5], and the formation of proteinase-K resistant α-syn deposits coincides with the presence of insoluble high-molecular weight α-syn species visible in native gel protein electrophoresis [43]. However, extensive aggregation does not occur unless seeding is induced by co-injection of α-syn fibrils [53]. One month after vector injection, we measure α-syn-induced changes in carbon incorporation as an indicator of neuronal dysfunction preceding overt neurodegeneration. A recent study using single-molecule Förster resonance energy transfer showed that A53T α-syn oligomerization occurs on intracellular membrane surfaces, in particular cardiolipin-rich mitochondrial membranes [10]. Although it is unclear where α-syn oligomers form in nigral neurons overexpressing α-syn, this finding is consistent with effects that we observe in the mitochondria and Golgi apparatus.

To understand how the mechanisms leading to neurodegeneration unfold inside neurons, we applied SILK-SIMS to characterize the initial effects of α-syn overexpression on cellular functions in vivo. We assessed anabolic activity inside nigral neurons by measuring the build-up of ^13^C labeling during a 48-h pulse of ^13^C-glucose administration, as well as the turnover of glucose-derived carbon in specific compartments during a 48-h chase period. Glucose is an essential fuel for the brain, where it contributes to major bioenergetic and biosynthetic pathways, including the glycolytic pathway, the pentose phosphate pathway, and mitochondrial metabolism [14]. Various glucose metabolites contribute to biosynthetic processes including glycogenesis in glia, lipid metabolism, amino acid synthesis, as well as the production of neurotransmitters, neuromodulators, and nucleic acids. While soluble metabolites are washed away during tissue processing applied in this study, NanoSIMS ^13^C imaging reveals the level of carbon incorporation into synthesized macromolecules including lipids, proteins and nucleic acids.

### Selective vulnerability of nigral dopaminergic neurons linked to high metabolism

Whereas the Lewy pathology characterized by α-syn deposition is not restricted to the SNpc in PD, this brain region contains dopaminergic neurons that are among the most vulnerable to the disease. Several arguments have been put forward to explain the selective vulnerability of nigral dopaminergic neurons, mostly related to intrinsic neuronal properties such as the production and metabolism of the dopamine neurotransmitter, which is linked to the production of reactive oxygen species and mitochondrial activity [23]. Nigral dopaminergic neurons have long, highly branched and unmyelinated axons that contribute to their high bioenergetic demand [6, 39, 44]. In addition, they display slow rhythmic spiking activity with large oscillations of intracellular Ca^2+^ that stimulate mitochondrial OXPHOS activity [50]. These features contribute in a cell-autonomous manner to metabolic and oxidative stress as well as impaired proteostasis, all together increasing the risk of neurodegeneration in conditions of energy deficiency [36]. Although high metabolic needs are considered as an important factor in the degeneration of specific neuronal populations, this aspect has rarely been addressed. Specific approaches need to be developed to allow for targeted analyses of cell metabolism in brain tissues. In the pulse-chase experiment with ^13^C-labeled glucose, NanoSIMS imaging of the nigrostriatal system reveals that large-sized neurons located in the SNpc display a strikingly higher level of glucose-derived carbon incorporation and turnover as compared to striatal neurons (Fig. 2). Although other neuronal populations will need to be investigated, nigral neurons appear to have high metabolic demand, as predicted from their morphological and electrophysiological properties.

### A method to assess changes in carbon incorporation and turnover following α-syn overexpression

Few techniques are available to assess macromolecule turnover at the cellular level in the mammalian brain. Using hippocampal neurons cultured with ^15^N-labeled leucine, combined NanoSIMS and fluorescence imaging showed a correlation between neuronal activity and presynaptic protein turnover [29]. NanoSIMS has also been used to assess ^15^N-leucine incorporation in hippocampal pyramidal neurons following erythropoietin administration in mice, as an indicator of protein synthesis [26]. To address neuropathology, SILK-SIMS analysis has mainly been used to determine the turnover of proteins implicated in neurodegenerative diseases, such as Aβ, tau and SOD1 and to analyze the dynamics of protein aggregation [35, 41]. NanoSIMS has also been used to map elements in the neuromelanin present in dopaminergic neurons of the human SNpc [4]. Here, we used NanoSIMS to assess the effects of locally induced α-syn overabundance on glucose-derived carbon incorporation and turnover in nigral neurons. Unilateral AAV6-α-syn injection induces α-syn overexpression in the SN, while the contralateral hemisphere injected with a non-coding vector is used as internal control for the SILK analysis. NanoSIMS imaging provides sensitive and high-resolution measurements of ^13^C enrichment and, combined with SEM-based segmentation of specific cell compartments, reveals changes in ^13^C labeling kinetics at a subcellular level. Although the nature of the ^13^C-labeled molecules is unknown, the ^13^C enrichments measured in tissue samples processed for SEM analysis reflect carbon incorporation into a broad suite of macromolecules in each cellular compartment, as a function of anabolic and catabolic fluxes. In order to take into account expected variations in ^13^C incorporation among animals and among individual neurons, values measured in each cellular sub-compartment were systematically related to the overall ^13^C level determined in the corresponding cellular compartment (cytoplasm and nucleus versus whole cell; organelles versus whole cytoplasm). When comparing control nigral neurons to those exposed to α-syn overexpression in the same animals, this revealed changes in the kinetics of ^13^C labeling and points to specific metabolic defects/anomalies, in particular in the mitochondria and Golgi apparatus.

### Effects of α-syn overexpression on neuronal ^13^C-glucose incorporation

Alpha-syn pathology can affect central metabolic pathways, such as lipid metabolism [1], by stimulating glucose uptake but impairing glucose metabolism [2, 46]. In the present study, the neurons located in the AAV6-α-syn injected hemisphere were characterized by a higher carbon turnover in the cell body during the chase period (Fig. 3c), indicating that the overall turnover of macromolecules might be increased in neurons overexpressing α-syn. In the rat SNpc, α-syn overexpression also leads to a redistribution of ^13^C labeling across the cytoplasmic and nuclear compartments. Whereas in the non-affected SNpc, neurons show higher ^13^C enrichment in the nucleus than in the cytoplasm during the pulse phase (Fig. 4d), there is no such difference between these two compartments following α-syn overexpression. Although multiple causes may account for this effect, one possible explanation is that α-syn can regulate nuclear activity by interacting with DNA and histones and regulate gene transcription [49]. Furthermore, α-syn has recently been found to affect the integrity of the nuclear envelope as well as the nucleocytoplasmic transport [9], to regulate the activity of processing bodies, and to stabilize mRNAs in the cytoplasm [25].

When analyzing specific organellar compartments, we find that α-syn overexpression leads to abnormally shaped mitochondria, as previously reported [18]. Furthermore, it induces a significant reduction in the level of ^13^C labelling in mitochondria during the pulse phase (Fig. 7b). This effect indicates alterations of mitochondrial metabolism, possibly related to the reported failure of mitochondria to import macromolecules as a consequence of α-syn interaction with the protein import machinery [13], or via the effects of α-syn at the level of mitochondria-ER contacts (MERC) [24, 40], an inter-organelle connection important for metabolic functions such as lipid metabolism [42]. Finally, at the level of the ER-Golgi axis, α-syn slows down macromolecule turnover in the secretory pathway, an effect revealed by elevated ^13^C levels in the Golgi apparatus at the end of the chase period (Fig. 7c). Overexpression of α-syn affects the ER-Golgi vesicular trafficking, which correlates with ER stress and Golgi fragmentation, and affects lysosomal glucocerebrosidase activity [12, 22, 33, 54]. In this context, overexpression of Rab1a can partially suppress α-syn toxicity [11, 12]. In addition to the early secretory pathway, α-syn can also disrupt intra- and post-Golgi secretory trafficking, as shown by its interaction with several Rab proteins [8, 11, 20, 56]. Overall, α-syn overexpression is a major perturbator of the secretory pathway [55], which is confirmed by our NanoSIMS imaging of glucose-derived carbon metabolism in the SNpc. Although we did not observe any significant changes in the ^13^C levels labeling of the lysosomal compartment following α-syn overexpression (Fig. 7d), we cannot exclude an effect of α-syn considering the broad variation in the ^13^C enrichment observed among individual lysosomes (Fig. 5c, d).

## Conclusions

This study illustrates how NanoSIMS can be used to reveal changes in carbon metabolism in specific cellular compartments and organelles following overexpression of α-syn, an abundant protein that has been shown to perturb inter-organelle molecular exchanges. NanoSIMS imaging provides sensitive imaging of stable isotope labeling with high spatial resolution. When correlated with SEM, this powerful approach can be used to reveal changes in the metabolic activity of neuronal subpopulations at the subcellular level and compare normal and disease conditions. Combined with various probes carrying specific isotopic labels (e.g., ^13^C or ^15^N) designed for specific biological processes, future nanoscale imaging using SILK-SIMS can help interrogate the dynamics of neuronal metabolism in the normal brain and investigate the metabolic changes underlying neurodegenerative diseases.

### Supplementary Information

**Additional file 1**. Correlating NanoSIMS images with ultra-high resolution EM images in Look@NanoSIMS.

## Data Availability

The datasets used and/or analyzed during the current study are available from the corresponding authors on reasonable request.

## Abbreviations

AAV: Adeno-associated virus
α-syn: Alpha-synuclein
ER: Endoplasmic reticulum
EsB: Energy selective backscatter detector
MERC: Mitochondria–ER contacts
PD: Parkinson’s disease
SEM: Scanning electron microscope
SILK-SIMS: Stable isotope labeling kinetics with secondary ion mass spectrometry
SNpc: Substantia nigra pars compacta
TU: Transducing units
