# Supplementary material for: Stable isotope labeling and ultra-high-resolution NanoSIMS imaging reveal alpha-synuclein-induced changes in neuronal metabolism in vivo
Source: Acta Neuropathol Commun. 2023 Sep 29;11:157. doi: 10.1186/s40478-023-01608-8 (PMC10540389; doi:10.1186/s40478-023-01608-8)
Supplement: Supplementary file 1 — Additional file 1. Correlating NanoSIMS images with ultra-high resolution EM images in Look@NanoSIMS. [file 40478_2023_1608_MOESM1_ESM.pdf]

Stable isotope labeling and ultra-high-resolution NanoSIMS imaging reveal alpha-synuclein-induced changes in neuronal metabolism *in vivo*

Sofia Spataro, Bohumil Maco, Stéphane Escrig, Louise Jensen, Lubos Polerecky,
Graham Knott, Anders Meibom, Bernard L. Schneider

Supplementary Information

1. Correlating NanoSIMS images with ultra-high resolution EM images in Look@NanoSIMS

NanoSIMS provides spatially resolved information about the isotopic composition of a sample, whereas electron microscopy (e.g., TEM or SEM) provides structural information. Because of the compatibility of these imaging techniques, both types of information can be obtained from the very same area of the sample. Here, we describe a workflow for performing correlative analyses of the images produced by NanoSIMS and EM in Look@NanoSIMS.

In this process, one needs to deal with the following issues:

a) **Considerably different pixel resolution.** Typically, the lateral spatial resolution in EM images is roughly 10-fold greater than in NanoSIMS images. For example, NanoSIMS images analyzed in this example have 256 × 256 pixels, whereas the image of the same sample area acquired via ultra-high-resolution EM has 4096 × 3072 pixels.
b) **Image shift and distortion.** Typically, NanoSIMS and EM images of the same sample area are *shifted* relative to each other via a combination of translation and rotation. This is caused by the difficulty of placing the sample exactly in the same orientation relative to the path of the rastering probing beam during the image acquisition in each instrument. Additionally, and more importantly, NanoSIMS and EM images are also *distorted* relative to each other. This is primarily because the NanoSIMS image acquisition is a lengthy process (ranging from tens of minutes up to several or even tens of hours) during which the sample stage can move on a scale of microns due to relatively minor temperature fluctuations in the laboratory hosting the instrument. Because the beam rastering and temperature fluctuations are uncorrelated, NanoSIMS images become somewhat distorted (e.g., a square image does not precisely correspond to a square area on a sample).

Because of these issues, the image processing workflow involved in the correlative analysis of NanoSIMS and EM images needs to account for image magnification, translation, rotation, and distortion.

The approach implemented in Look@NanoSIMS to deal with these effects has two components. First, the NanoSIMS ion count data are *resampled* such that each pixel in the original NanoSIMS image is transformed into a square area with M × M pixels in the modified NanoSIMS image, whereby the ion counts per pixel in the square area are, for each detected mass, decreased by a factor of M^2 relative to the counts in the original pixel. Effectively, this resampling yields an M-fold magnified image with an M^2-fold increased number of pixels and M^2-fold decreased ion counts per pixel. This transformation is done using Matlab's function `imresize`. To ensure that the sequence of forward (by a factor M) and backward (by a factor 1/M) magnification yields exactly the same ion count data, image resizing is done using the `nearest` method. Furthermore, the factor M, which can be specified by the user via a dialog box, is constrained such that M is an integer when M>1 and 1/M is an integer when 0<M<1.

The second step in the workflow is based on the definition of multiple pairs of reference points (typically >4, the more the better) that correspond to each other in the resampled NanoSIMS and EM images. Using the coordinates of these corresponding pairs of reference points, Matlab's functions `fitgeotrans` and `imwarp` are combined to map the EM image onto the resampled NanoSIMS image.

Once these two steps are performed, the transformed EM image can be imported into Look@NanoSIMS and used as an additional 'ion count image' (named `ext`) to carry out further data processing steps, such as drawing of regions of interest (ROIs) based on the EM image, exporting of NanoSIMS and EM image overlays, exporting of ROI-specific ion count ratios, etc.

Below, we describe the step-by-step workflow for the correlative analysis of NanoSIMS and EM images in Look@NanoSIMS. We use the images shown in **Figure 1a-b** as examples (data available at URL) and assume that the main Look@NanoSIMS graphical user interface (GUI) is opened in Matlab.

1.1 *Import and visualize raw NanoSIMS image data*

- Click on Input → Load RAW dataset and choose an `.im` or `.im.zip` file (in this example we use the file `Spataro-Sept-2018_3.im`).
- Click on Input → Autoscale plane images.
- Click on Input → Display plane images for all masses. In the dedicated GUI, click on the arrows (|<, <<, <, >, >>, >|) to explore the ion counts images for all detected masses.

1.2 Alignment and accumulation of planes

- In the Accumulation options box, specify Base mass for alignment (here 14N12C) and check the Align images when accumulating checkbox.
- Click on Input → Display alignment mass. In the dedicated GUI, click on Define alignment region to define the region based on which plane alignment will be determined. Click Close when done.
- Click on Input → Accumulate plane images. Observe the progress of image alignment and accumulation in Matlab's console.
- Click on Input → Autoscale accumulated images.
- Click on Input → Display accumulated images for all masses.

1.3 Resampling of the NanoSIMS images

This is the first critical step in the correlative analysis: modifying the pixel resolution of the NanoSIMS images to (roughly) match the pixel resolution of the EM image. This step requires the corresponding resolutions to be known.

We emphasize here that resampling by a factor of M changes the amount of NanoSIMS data M^2-fold. Thus, choosing too large a value for M might cause instability of the Matlab session, or even of the computer, if the available RAM is insufficient to handle this. Thus, it is wise to make a trade-off between resolution matching and computer performance.

In this example, we know that the NanoSIMS images have 256 × 256 pixels, whereas the EM image of roughly the same sample area has 4096 × 3072 pixels. Thus, we choose the value of 10 for the magnification factor M. This value is also good considering that, in the very end, we may want to scale the

NanoSIMS and ROI images back to the original size. For this back-transformation we will need to use the factor M = 0.1, which is allowed because 1/0.1 = 10 is an integer.

- Click on Input → Change resolution of accumulated masses and type in 10 for the magnification factor to **resize the NanSIMS data 10-fold**. Carefully read the messages in the dialog windows.
- Click on Input → Autoscale accumulated images.
- Click on Input → Display accumulated images for all masses.
- Verify that the correct resizing was performed. The current size of the NanoSIMS images appears at the bottom right of the displayed image (in this example, [resized: 2560x2560 pix]).
- Click on Output → Display masses to display the images of the resampled ion counts. This step is important because it generates a mat file for each detected mass (e.g., the file `14N12C.mat` for the $^{14}N^{12}C$ ion counts), which is important for the subsequent steps. Note that the resampled images look the same as for the original data (before resampling), except that the number of pixels increased 100-fold and the ion counts are 100-fold lower.
- Click on Output → Display ratios to display the images of the resampled ion count ratios. Similar to the ion count images, the images look the same before and after resampling.

1.4 Alignment of the NanoSIMS and EM images

This is the second critical step in the correlative analysis: mapping of the EM image onto the resampled NanoSIMS image. This step requires that distinct features visible in the EM image can be identified in at least one of the NanoSIMS images. Positions of these features will be manually defined in both the EM and NanoSIMS images.

- Click on External → Align external and nanosims images.
- In the dedicated GUI, click on File → Load external image and select the external image (in this example the file `Rat7SNR_21.tif`). Note the image size using the annotation of the x and y axes.
- Click on File → Load nanosims image and select the nanosims mass image (for example the `14N12C.mat` file generated previously; see Section 1.3 above). Using the annotation of the x and y axes, verify that the loaded image corresponds to the resampled data (here 2560 × 2560). After this step, the GUI may look as in the following example.

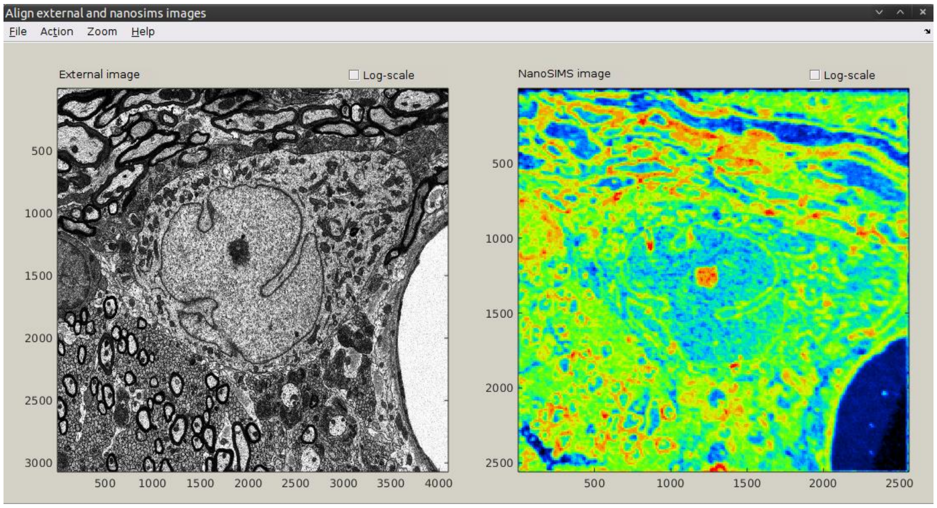

- Click Action → Add point to define a reference point in the EM image. Note the text displayed in the Matlab’s console for useful tips (e.g., left-mouse click for selecting the point location, arrows for fine-tuning, right-mouse click or “Enter” for confirming the point location).
- Click Action → Add point to define the corresponding reference point in the NanoSIMS image.
- Repeat the previous two steps to define multiple reference point pairs (the more, the better).
- Use the Action menu to refine or remove reference points until you are satisfied. After these steps, the GUI may look as in the following example.

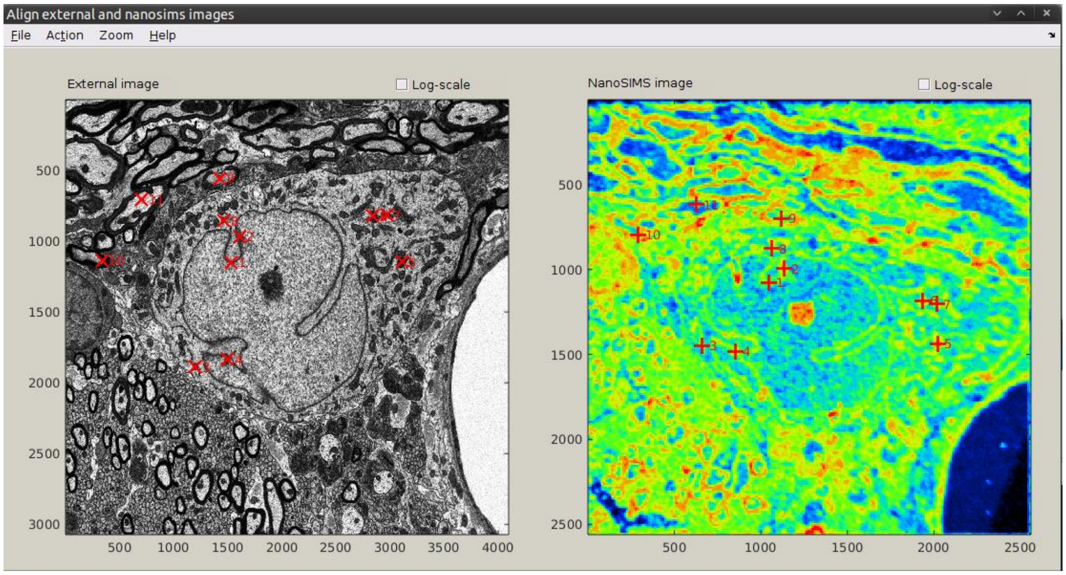

- Click on File → Save point list and specify the output file name (e.g., `points_10x.mat`). It is useful to add information about the magnification factor to the file name for future reference.
- Click on Action → Alignment based on N>4 points to **perform the image alignment**. The result may look as shown in the following example. Note how the reference points defined for the EM and NanoSIMS images are mapped onto each other.

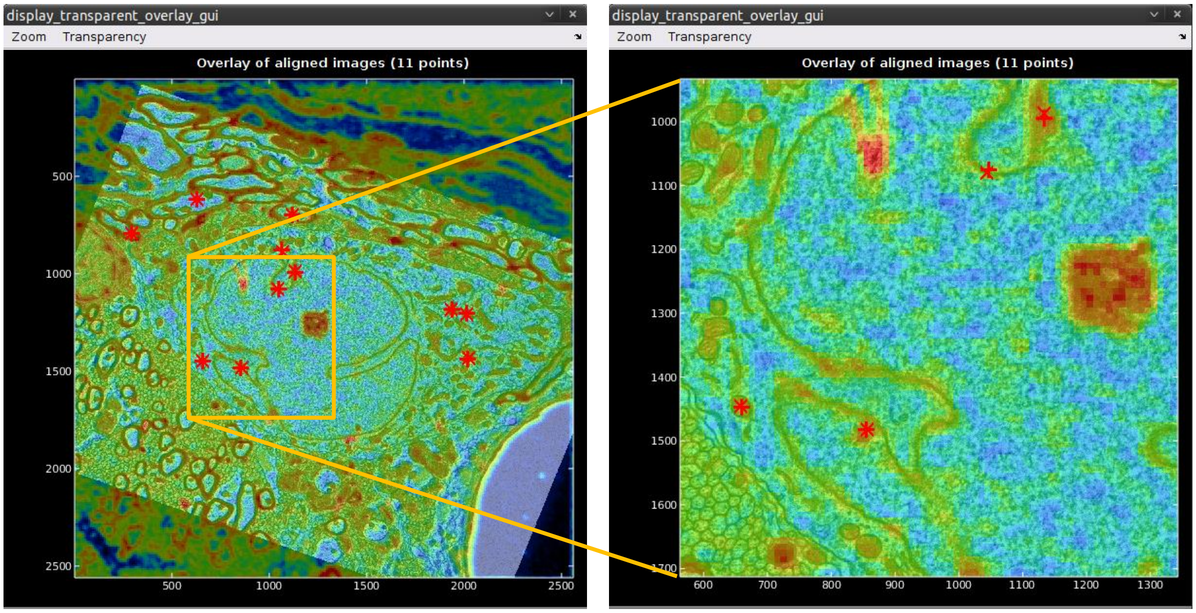

- Use actions in the Zoom and Transparency menus to verify the quality of the alignment.
- When satisfied with the alignment quality, click on Action → Export aligned external image, choose an output file name (e.g., `EM_aligned_10x.mat`, best in the `mat` subfolder), and click Save. Note that appending the magnification factor to the filename (`_10x`) helps remembering this value when using the aligned EM image in future analyses.

1.5 Adding aligned EM image to the NanoSIMS image set

- In the main GUI of Look@NanoSIMS, click on External → Select aligned external image. Select the mat file exported in the previous step (e.g., `EM_aligned_10x.mat`) and click Open.

From this moment onwards, data from the aligned EM image are accessible within Look@NanoSIMS as one of the accumulated ion count images. The name of the "mass" is `ext`, and the identification number is N+1, where N is the number of masses in the NanoSIMS image set (in this example, N=8 and thus the ID of the aligned EM image is 9).

1.6 Overlay of NanoSIMS and EM images

One of the reasons for importing the ultra-high-resolution EM image into Look@NanoSIMS is to visually inspect overlays between the EM image and the various ion count images acquired by NanoSIMS. In the following example we export an RGB overlay between the $^{14}N^{12}C$ ion count image (as the green channel of an RGB image) and the inverse of the EM image (as the blue channel).

- In the main GUI, type `14N12C` in the text area for the ratio expression #4 and specify the corresponding scale.
- Type `1-ext` in the text area for the ratio expression #3 and specify the corresponding scale.

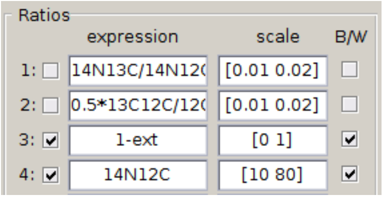

- Check the Display images, Combine images as RGB, and Pixel by pixel checkboxes in the Output options box, and type 4 and 3 into the text areas for the green (G) and blue (B) channel, respectively.

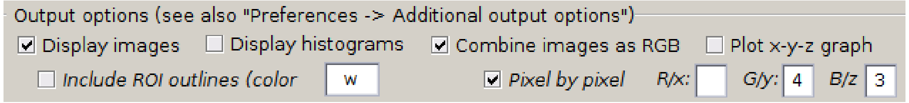

- Choose Output -> Display ratios. The results should look similar to the following example.

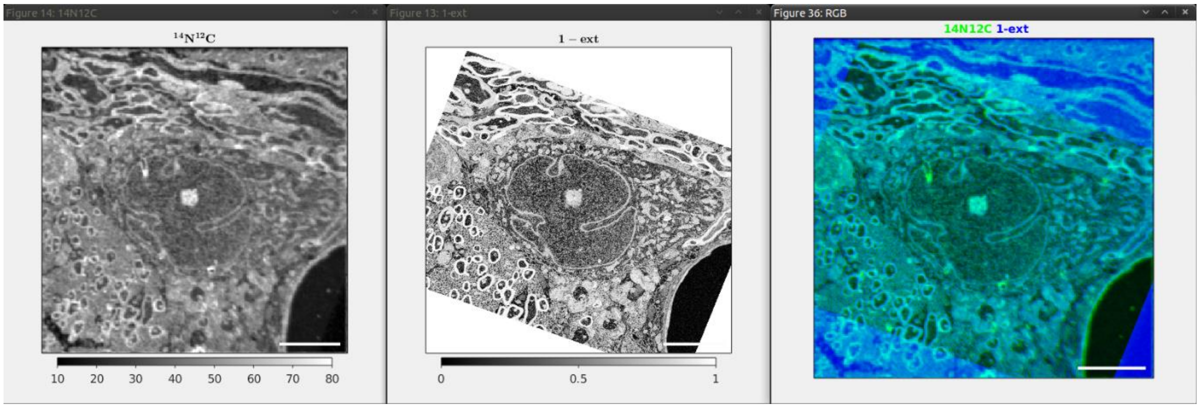

1.7 Using aligned EM image to define ROIs

Another reason for importing the ultra-high-resolution EM image into Look@NanoSIMS is to precisely define ROIs corresponding to, e.g., intracellular organelles. In this example, we use the aligned EM image as the template for ROI definition.

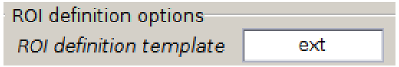

- In the main GUI, type `ext` in the text area for the ROI definition template in the ROI definition options box.
- Click on ROIs → INTERACTIVE ROIs definition tool.
- In the dedicated GUI, use the Action menu to define ROIs. For example, use Draw ROI freehand or Interactive thresholding for manual or semi-automated ROI definition, respectively. Note useful tips in the Matlab's console. Couple of ROIs defined in this way are shown in the following example, including the full-scale image and a zoomed-in area.

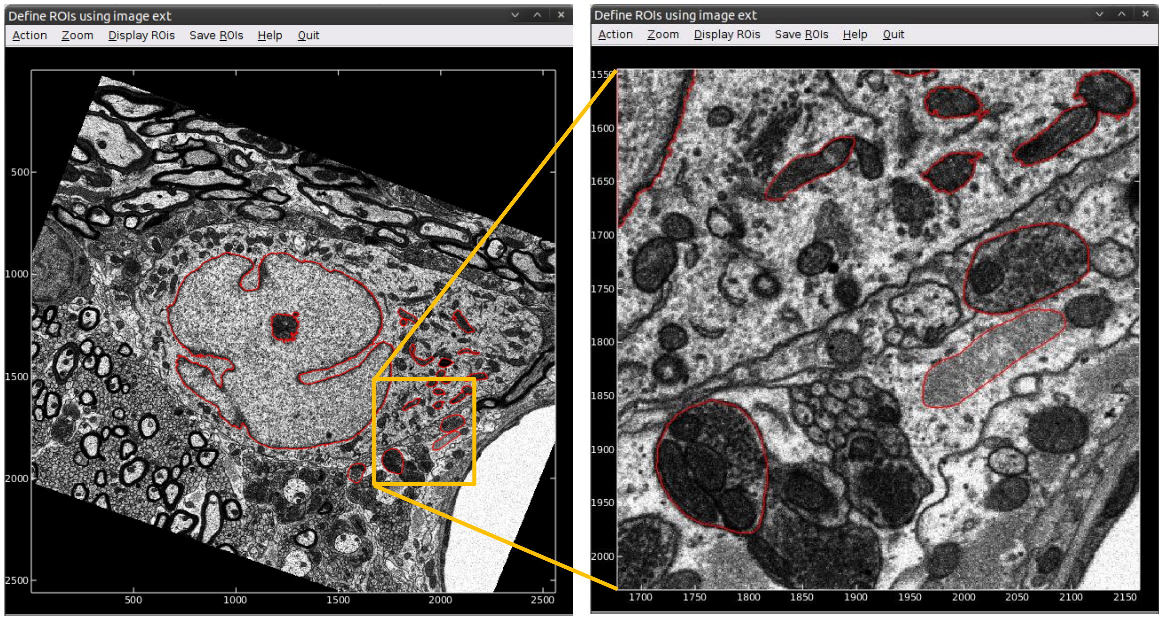

- Click Display ROIs → Display ROIs with ROI ID's to show the currently defined ROIs together with their identification numbers. Note that when you zoom in the image, the ROI outlines are smooth, which is because of the high resolution of the image used to draw them. The result may look similar to the following example.

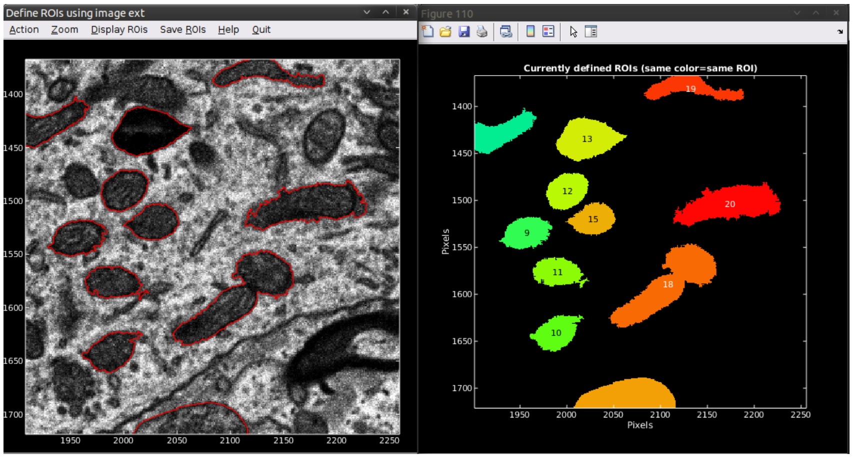

- Click on Save ROIs → Save ROIs, choose an output file name (e.g., `ROIs_10x.mat`), and click Save to save the currently defined ROIs. Note that appending the magnification factor to the filename (e.g., `10x`) helps keeping you alert that the file `ROIs_10x.mat` stores ROIs defined for the 10-fold magnified NanoSIMS images, which will be useful in future analyses.
- Click on Display ROIs → Display magnification-corrected ROIs to show how the currently defined ROIs will look when resampled to the original dimensions of the NanoSIMS images. Because in this example the original dimensions are 10-fold smaller, the ROI outlines will look much *coarser* when you zoom in the image, as shown in the following example.

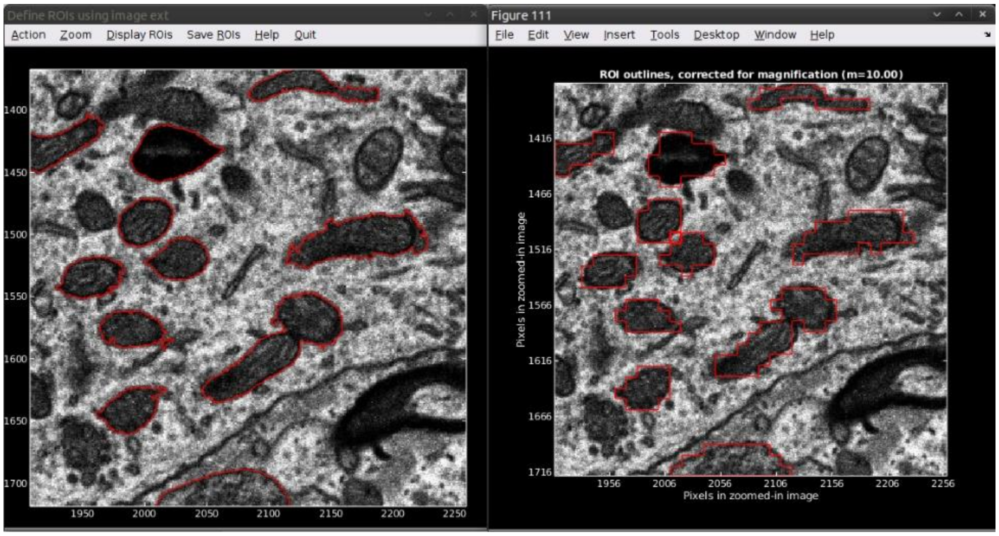

- Click on Save ROIs → Save magnification-corrected ROIs, choose an output file name (e.g., `ROIS_1x.mat`), and click Save to save the ROIs. Again, appending the magnification factor to the filename (`1x`) helps keeping you alert that the file `ROIs_1x.mat` stores ROIs defined for the *original* (i.e., unmagnified) NanoSIMS images.
- Close the ROI definition GUI once you have defined and saved the ROIs.

1.8 Quantifying ion count ratios in ROIs

The defined ROIs can be used to estimate ROI-specific isotope ratios from the ion count data. For example, the $^{13}C/^{12}C$ ratio can be estimated as the $^{14}N^{13}C/^{14}N^{12}C$ ion count ratio. There are two options to do this: using either the resampled (e.g., magnified) or original NanoSIMS images. The results differ from each other because the former option uses fractions of ion counts in pixels at the ROI periphery, which is not possible when the ROIs are resampled from a higher to a lower resolution. The differences are small for relatively large ROIs, i.e., when the number of pixels at the ROI periphery is small compared to the total number of pixels per ROI. Nevertheless, it is important to be aware of these differences and the underlying reason.

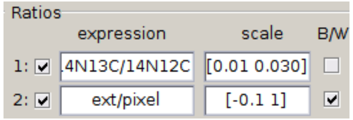

In the following example, we calculate the ROI-specific $^{14}N^{13}C/^{14}N^{12}C$ ion count ratios and plot them against the average brightness of ROIs as they appear in the EM image. To achieve this, type `14N13C/14N12C` and `ext/pixel` in the text area for the ratio expression #1 and #2, respectively, and specify the corresponding scales.

Additionally, check the Plot x-y-z graph and ROI-averaged checkboxes and type 2 and 1 to the R/x and G/y text areas, respectively. Also ensure that the checkbox Export ASCII data is checked.

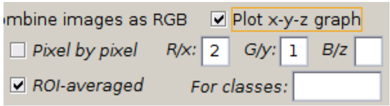

Follow these steps to quantify the ion count ratios using the *resampled* NanoSIMS images:

- Click on Input → Autoscale accumulated images and Input → Display accumulated images for all masses to verify that the NanoSIMS images have been resampled. In this example, this is indicated in the lower-right corner of the displayed image [resized: 2560×2560 pix].
- Click on ROIs → Load ROIs from disk, select the file name corresponding to ROIs defined previously using the resampled NanoSIMS data (e.g., `ROIs_10x.mat`), and click Open.
- Click on ROIs → Export ROIs image as graphics.
- Check the Display images, Include ROI outlines, and Export PDF graphics checkboxes in the Output options box (main GUI).
- Click on Output → Display ratios. Results may look as shown in the following example.

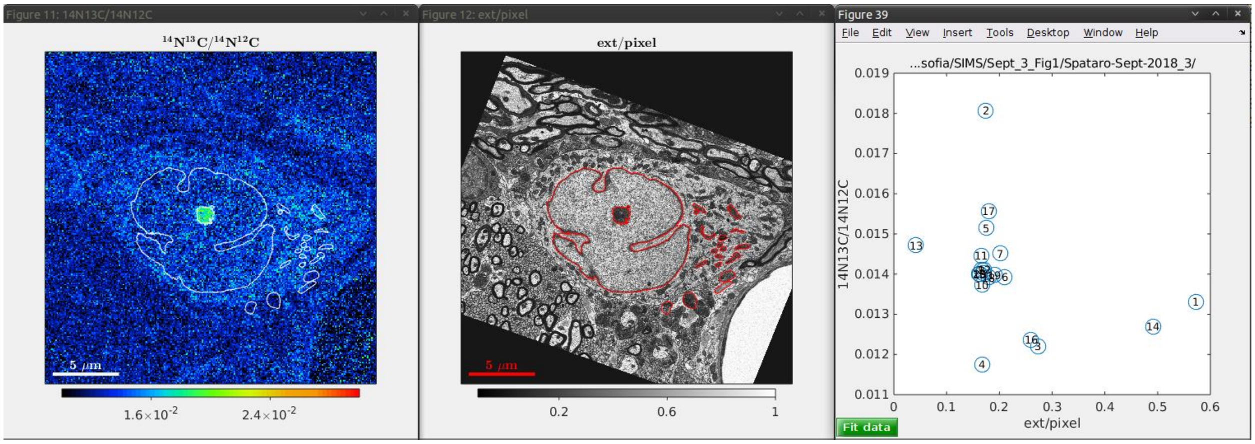

- The ROI-specific ratio values are exported in a dac file located in the dat subfolder (e.g., `14N13C-14N12C.dac`, `ext-pixel.dac`).
- The images are exported in the pdf subfolder (e.g., `14N13C-14N12C.pdf`, `ext-pixel.pdf`).

Follow these steps to quantify the ion count ratios using the *original* NanoSIMS images:

- If the data has been previous resampled to a higher resolution, they need to be resampled back to the original resolution. This is achieved by clicking on Input → Change resolution of accumulated masses and typing a value lower than 1 for the magnification factor M (0.1 in the example shown here).
- Click on Input → Autoscale accumulated images and Input → Display accumulated images for all masses to verify that the NanoSIMS images are *not* resampled. This is the case if the [resized: … pix] string is *missing* in the lower-right corner of the displayed image.
- Click on ROIs → Load ROIs from disk, select the file name corresponding to ROIs for the *original* NanoSIMS data (e.g., `ROIs_1x.mat`), and click Open. Recall that this file was saved in the step Save magnification-corrected ROIs, as described above (Section 1.7).
- Click on ROIs → Export ROIs image as graphics.

- Check the Display images, Include ROI outlines, and Export PDF graphics checkboxes in the Output options box (main GUI).
- Click on Output → Display masses to display the original ion counts images. This step generates a mat file for each detected mass (e.g., the file `14N12C.mat` for the $^{14}N^{12}C$ ion counts). Note that this action overwrites the mat files generated when the NanoSIMS data was resampled (see Section 1.3). Also, the files will be roughly 100-fold smaller because of the 10-fold decreased resolution.
- Click on Output → Display ratios. Results may look as shown in the following example. Note the coarser appearance of the `ext/pixel` image and a slightly different values of the ROI-specific ion count ratios in the scatter plot. This is not surprising, as described at the beginning of this section.

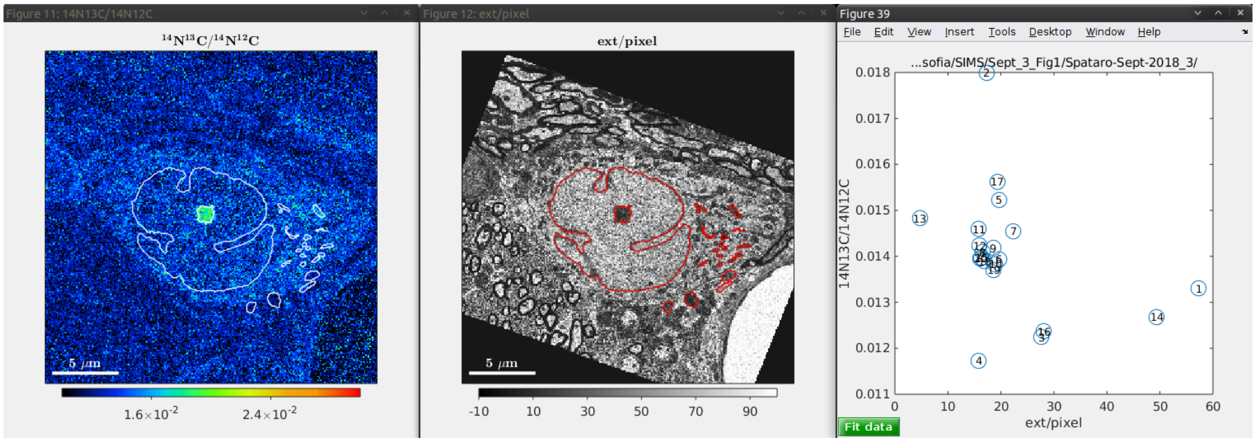

- The data and images are exported in the corresponding dat and pdf subfolders, respectively. Note that this action will overwrite the dac files generated previously for the resampled NanoSIMS data. It may be a good idea to keep a backup of those files for comparison.

1.9 Finishing the analysis

- Click on External → Select aligned external image and then click Cancel to *remove* the EM image from the NanoSIMS dataset. From this moment, `ext` (i.e., "mass" with an identification number N+1) will no longer be available within Look@NanoSIMS.
- Click on Preferences → Store preferences, select an output file name (e.g., the default `prefs.mat`), and click Save to store the settings of the Look@NanoSIMS session corresponding to the current dataset.
- Click on Preferences → Backup folder with processed data to create a backup of the most important files generated during the analysis (i.e., alignment of planes, reference points for the alignment of NanoSIMS and EM images, ROIs defined for the resampled and original datasets, preferences).
